# Exploring the Mechanism of Danshen against Myelofibrosis by Network Pharmacology and Molecular Docking

**DOI:** 10.1155/2018/8363295

**Published:** 2018-12-05

**Authors:** Jie Li, Xiaoran Ma, Cun Liu, Huayao Li, Jing Zhuang, Chundi Gao, Chao Zhou, Lijuan Liu, Kejia Wang, Changgang Sun

**Affiliations:** ^1^College of First Clinical Medicine, Shandong University of Traditional Chinese Medicine, Jinan 250014, Shandong, China; ^2^College of Traditional Chinese Medicine, Shandong University of Traditional Chinese Medicine, Jinan 250014, Shandong, China; ^3^Department of Oncology, Weifang Traditional Chinese Hospital, Weifang 261041, Shandong, China; ^4^College of Basic Medicine, Qingdao University, 308 Ningxia Road, Qingdao 266071, Shandong, China

## Abstract

Danshen (Salvia miltiorrhiza Bunge), a natural powerful drug for various conditions treatment, has traditionally been used in Asian countries for centuries as anticancer agent, anti-inflammatory agent, and antioxidant. More recently, it is explored in combination with other herbs for skeletal diseases therapy; bone-targeting compounds with pharmacological activities have been isolated from various sources of traditional Chinese medicine (TCM), including Danshen. In this case, some evidence supports that Danshen may treat myelofibrosis (MF) by exerting its antitumor effect. To study the specific mechanism of Danshen in the treatment of MF, we used bioinformatics databases to determine its active ingredients. Then, identification of target proteins related to MF was made using a network pharmacology analysis platform. In our results, 20 key active compounds and 457 key targets of Danshen were identified. In-depth network analysis of the top diseases, functions, and pathways suggested that a common underlying mechanism linked Danshen involvement with MF. Finally, 5 potential targets were confirmed by the analysis; these 5 targets, as well as 20 previously identified compounds, were subjected to molecular docking experiments. The results indicated that cryptotanshinone of Danshen may affect MF by acting on the key genes in the JAK-STAT signalling pathway and the TGF-*β* signalling pathway.

## 1. Introduction

Myelofibrosis (MF) is a clonal stem cell disorder classified as a chronic myeloproliferative neoplasm (MPN). It is characterized by extensive bone marrow fibrosis, overproduction of inflammatory cytokines, progressive splenomegaly, and anaemia [1]. MF impacts quality of life and survival. At present, hematopoietic stem cell transplantation (HSCT) remains the only way to cure MF [2]. However, HSCT has apparent limitations for most patients. It has been proved that overactive JAK/STAT is an iconic signalling pathway for MF. The therapy of MF currently is ruxolitinib, which can recognize driver mutations of JAK2, improve splenomegaly, and alter the disease course further, prolonging survival in some patients [3, 4]. Nevertheless, ruxolitinib is limited by drug resistance. Furthermore, some studies revealed that proinflammatory cytokines appear to show good effects as promising therapies [5]. Recently, many pharmacologic treatments related to JAK1/2 and cytokine-cytokine receptor interactions have given rise to increased attention. In particular, regarding natural products, many patients are more inclined to seek complementary and alternative medicine [6].

Danshen (Labiatae sp. plant, the dried root of Salvia miltiorrhiza Bunge) is a common and important Chinese medicine with well-defined phytochemicals. As a type of complementary and alternative medicine, it has been used widely for a long time and shown significant clinical outcomes in terms of anticoagulant, antioxidant, and anti-inflammatory activities to benefits for vascular protection and cancer treatment [7–10]. The major biologically active components of Danshen include water-soluble phenolic acids and lipophilic tanshinones. Among them, cryptotanshinone and tanshinone IIA are the most abundant diterpenoids of tanshinones. Phenolic acid has antioxidant and anticoagulant activities, while tanshinone has antibacterial, antioxidant, and antitumor activities [11]. In terms of traditional Chinese medicine theory, Danshen cools the blood, eliminates carbuncles, soothes the nerves, promotes blood circulation by removing blood stasis, regulates menstruation, and alleviates pain. In past traditional studies, Danshen was mostly used for treating cardiovascular and cerebrovascular diseases. It has been reported that more than 900 Danshen preparations for various diseases are available in China, particularly for cardiovascular and cerebrovascular disorders [7, 8]. It also has reports indicating that Danshen has excellent anticancer effects, including inhibition of proliferation, restraint of angiogenesis and metastasis, and circumvention of multidrug resistance [9]. For example, Sumiyasuren Buyanravjikh et al. [10] found that cryptotanshinone (CRT), a natural product extracted from Danshen, effectively decreased secretion of proinflammatory cytokines including interleukin-1*β* and tumor necrosis factor-*α*. Furthermore, Danshen inhibited hyperproliferative fibroblasts, according to a report [12]. This suggests that Danshen is likely to act on myelofibrosis via some route; however, its mechanism has not yet been fully elucidated.

As a type of complementary and alternative medicine, herbal medicine has the advantage of low side-effects, low resistance, and long actuation duration. However, traditional Chinese medical science is composed of several components, targets, and pathways that realize its distinctive therapeutic efficacy by modulating human biological networks in body systems [13]. It is relatively difficult to clarify the mechanism of disease treatment accurately through traditional experimental methods. Thus, it is necessary to find an innovative measure for explaining pharmacological effects and mechanisms. With the rapid development of bioinformatics, the network pharmacology approach provides a great choice for revealing herbal medicine's molecular mechanisms efficiently and systemically [14]. It can take drugs and targets to abstract to a network model, and then show the intrinsic relationships of drugs and the relationships of targets in a network fashion, especially with respect to interactions and interrelationships as a whole [15].

Traditional Chinese medicine believes in holism; that is, Chinese herbs are used to treat diseases through several components and several targets rather than single components and single targets. The holistic perspective reflected in network pharmacology is perfectly in harmony with the concept of holism in TCM. In addition, other bioinformatics resources including molecular docking software have been exploited, providing good opportunities for rapid system filtering [16, 17]. It contains a well-designed function of scoring molecular docking to assess protein-ligand binding potential based on network pharmacology prediction and analysis [18]. Andrew L Hopkins believes that the network pharmacology strategy is a modern transformation of the initial and highly successful drug discovery method of Paul Janssen [19].

**Figure 1:**
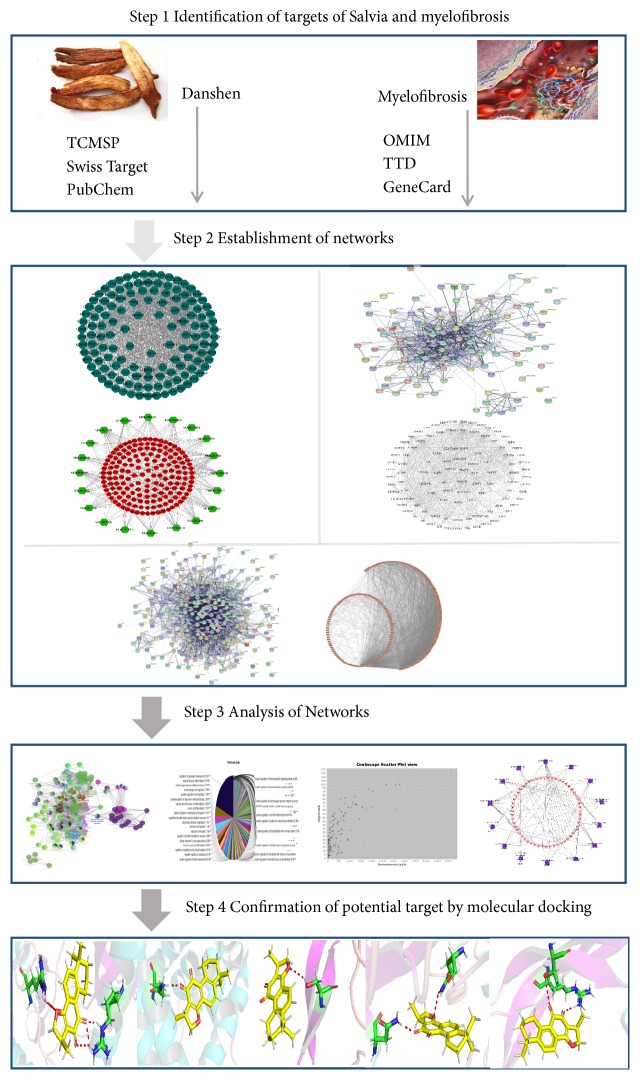
Workflow for Salvia treatment of myelofibrosis.

Therefore, in this study, a pharmacology network and molecular docking combination approach was introduced to explore the impact of Danshen on MF, as well as its molecular mechanisms. It is expected to provide reliable essential information and the feasibility of Danshen in treatment, probably as a potential therapy for MF. The work involved the following four steps: (1) the ingredients of Danshen along with their corresponding targets and myelofibrosis associated targets were identified by various databases; (2) the interaction relationships in the compound, the compound target, the disease target, and the function and pathways were constructed by networks; (3) the key compound, the key targets, the top functions, and the top canonical pathways were revealed by network analysis; (4) the most potentially valuable targets were obtained and validated through scoring between the top targets and corresponding compounds; and (5) the feasibility of the targets that may be the potential target in treatment of myelofibrosis was compiled by document retrieval. The workflow is displayed in Figure 1.

## 2. Material and Methods

### 2.1. Chemical Compounds in Danshen

We collected the chemical compound data of Danshen from the Traditional Chinese Medicine Systems Pharmacology Database (TCMSP, http://lsp.nwu.edu.cn/), which is a unique system pharmacology platform devised for Chinese herbal medicines. To discover the active components of Danshen, we chose compounds meeting the requirements of both OB ≥ 50% and DL ≥ 0.18. Then, 20 compounds in Danshen were obtained. OB represents the percentage of an orally administered dosage of unchanged drug that reaches the systemic circulation, indicating the convergence of the ADME (absorption, distribution, metabolism, and excretion) process. DL has been widely used to filter out compounds with undesirable properties.

### 2.2. Compound Targets for Danshen

To obtain putative targets of potential active compounds in Danshen, we used the SwissTargetPrediction (http://www.swisstargetprediction.ch/) and the TCMSP database. We input all the active compounds into PubChem (https://pubchem.ncbi.nlm.nih.gov/), obtained the canonical SMILES strings of active compounds in Danshen, and imported these canonical SMILES into Swiss target to obtain targets of each compound. Finally, we used the UniProt KB search function (http://www.uniprot.org/) in the UniProt database, entered the protein name, and defined the species as “human”, in order to obtain the gene name for each protein. Eventually, 457 compound targets for Danshen were retrieved without repeating targets of the same compound.

### 2.3. Potential Target Genes of Myelofibrosis

Information regarding MF-associated target genes was gathered from the Online Mendelian Inheritance in Man database (OMIM: https://www.ncbi.nlm.nih.gov/omim), the therapeutic targets database (TTD; http://bidd.nus.edu.sg/BIDD-Databases/TTD/TTD.asp) and the human gene database (GeneCards; http://www.genecards.org/). Only ‘Homo sapiens' proteins related to myelofibrosis were selected.

### 2.4. Network Construction and Analysis

To evaluate potential protein interactions among the targets, all gene symbols of the protein targets including Danshen and MF were submitted to the String Database (https://string-db.org). Only protein interactions with the confidence score 0.400 were considered. We inputted these targets and the data into the network visualization software Cytoscape (http://cytoscape.org/, ver. 3.6.0) to construct the 5 following networks: (1) compound target network; (2) compound-compound target network; (3) disease target network; (4) compound target-disease target network; and (5) disease target-pathway network. Among these networks, topology analysis was achieved in CentiScaPe.

### 2.5. Gene Ontology Enrichment and Pathway Analysis

ClueGo was applied for Gene Ontology (GO) enrichment analysis, and the biological processes with P-value <0.05 were considered significant biological processes. Potential targets were uploaded to the Database for Annotation, Visualization and Integrated Discovery (DAVID, https://david.ncifcrf.gov/, ver. 6.8), and KEGG pathway information was obtained. Only terms and enrichment pathway with P-value <0.05 were considered and thought to construct the disease target-pathway network.

### 2.6. Molecular Docking between Targets and Compounds

All small molecule structures of the 20 compounds in our previous work were obtained from the PubChem Project and were saved to mol2 format files. All protein-ligand complexes with crystal structures of the above-mentioned 5 targeted proteins were directly obtained from the RCSB Protein Data Bank (http://www.rcsb.org/pdb/home/home.do, last accessed Dec 27, 2016). For improving the accuracy of results, the conduct of docking used SYBYL-X software and systemsDock (http://systemsdock.unit.oist.jp/iddp/home/index/) [20]. SYBYL-X, as well as systemsDock, is based on network pharmacology prediction and analysis that allows docking simulations and molecular pathway maps to fully characterize ligand selectivity and to interpret the role of ligands in complex molecular networks [18]. After the preparation work of adding hydrogenation as well as removing cocrystallized ligands and water molecules from the protein-ligand complexes, SYBYL-X and systemsDock were used to continue docking. The docking scores between these compounds and target proteins were used as the evaluation criteria to further screen out potential active components and to validate the potential targets. Combined with the results of both, the targets with docking scores greater than 6.0 were thought to be meaningful [18, 21].

## 3. Result

### 3.1. Identification of Targets of Danshen and Myelofibrosis in Various Databases

A total of 202 compounds from Danshen were selected from the TCMSP database, and active components were evaluated using DL and OB approaches (OB≥ 50%, DL≥ 0.18). Twenty active compounds (Table S1) were ultimately chosen for further investigation. From the Swiss target and TCMSP database results, we obtained potential targets for all 20 active compounds (Table S2). Deleting duplicate targets of the same compound between the two databases, 457 targets were identified for 20 compounds of Danshen. Meanwhile, using three various databases, 114 disease genes with no repeats were acquired (Table S3).

### 3.2. Establishment and Analysis of Several Networks

**Figure 2:**
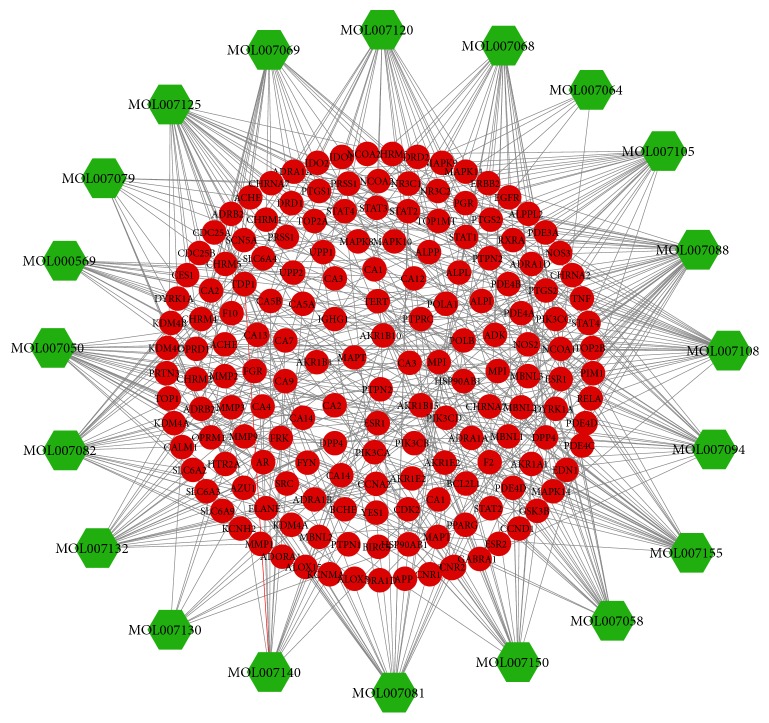
Compound-compound target network (green hexagons represent compounds contained in Salvia, and red circles represent compound targets).

**Figure 3:**
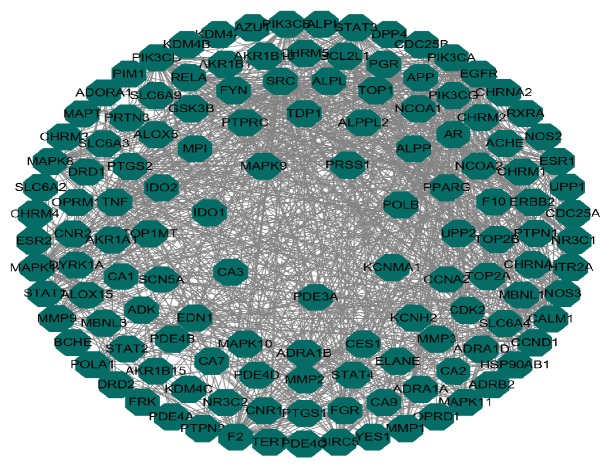
Compound target network (hexagons represent compound targets).

**Figure 4:**
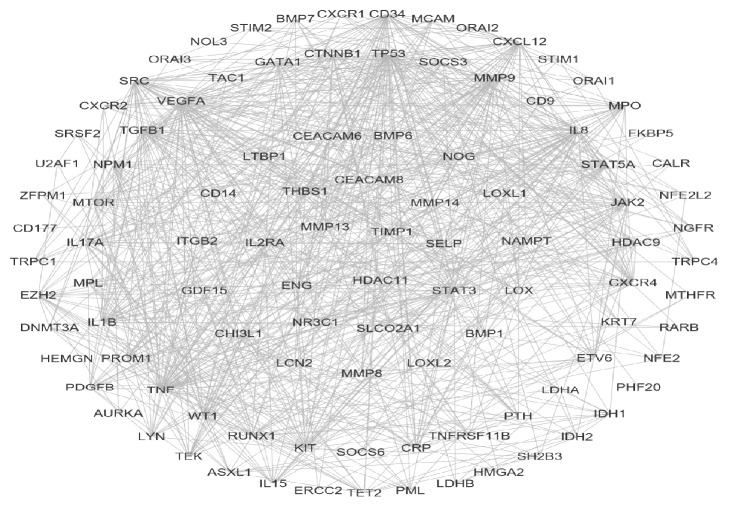
Disease target network (rectangles represent disease targets).

**Figure 5:**
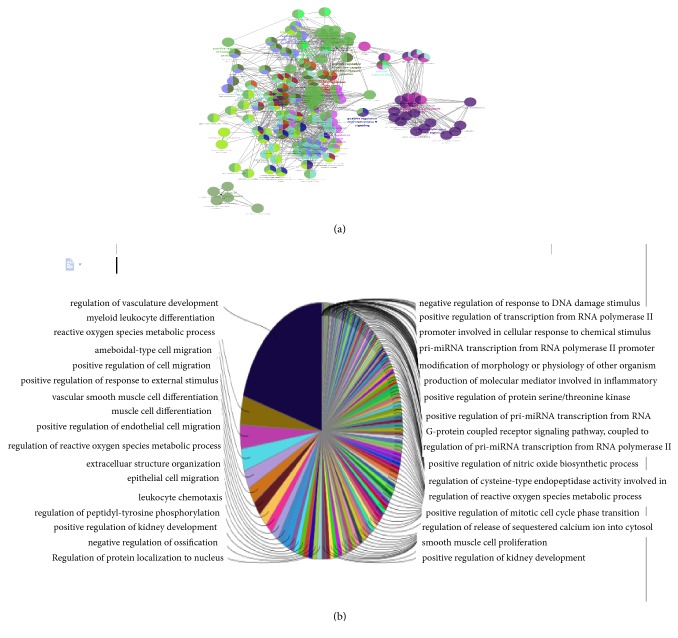
The GO enrichment analysis is represented by the pie charts (b), as generated by ClueGo, and the most vital term in the group is labeled (a).

**Figure 6:**
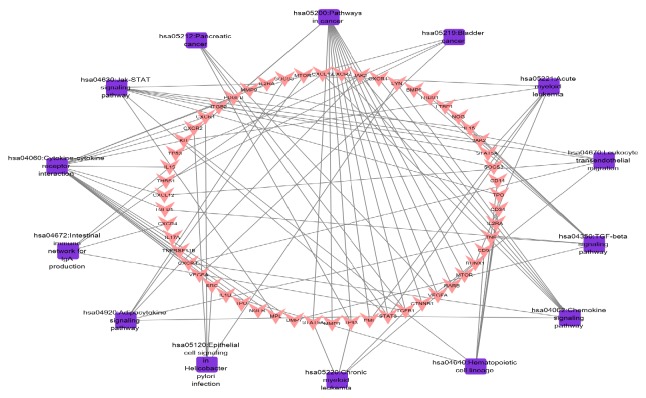
Disease target-pathway network (purple squares represent pathways, and red inverted triangles represent disease targets).

For displaying intuitive interactions in Danshen, compound-compound target network as shown in Figure 2 contained the 20 ingredients modulating 169 targets (189 nodes and 456 edges). The compound target network is depicted in Figure 3, including 133 nodes and 1060 edges. The disease target network in Figure 4 has 103 nodes and 731 edges. To define the precise relationship of Danshen and MF, a compound target-disease target network was built including 120 compound targets and 114 disease targets (234 nodes and 2818 edges). All targets were first filtered by String in order to remove duplicates. The results of topology analysis showed that 20 nodes with Degree Undir >65 could be considered as preliminary major nodes, including TP53, SRC, TNF, VEGFA, PIK3CA, IL8, EGFR, STAT3, PIK3CG, PIK3CD, PIK3CB, PTGS2, ESR1, TGFB1, CXCL12, CCND1, JAK2, TNNB1, MMP9, and CALM1. Then, the above genes in the top ten of the Betweenness Undir or Closeness Undir were screened out as follows: TP53, TNF, VEGFA, STAT3, and JAK2. The detailed score information is shown in Tables S4 and S5. Utilizing Go and KEGG, screened by the standard of P-value <0.05, we obtained 14 pathways, including cytokine-cytokine receptor interaction, pathways in cancer, hematopoietic cell lineage, the JAK-STAT signalling pathway, the TGF-*β* signalling pathway, the chemokine signalling pathway, acute myeloid leukaemia, the adipocytokine signalling pathway, leukocyte transendothelial migration, bladder cancer, intestinal immune network for IgA production, epithelial cell signalling in Helicobacter pylori infection, pancreatic cancer, and chronic myeloid leukaemia. The path lists are shown in Table S6. The results of GO analysis are displayed in Figure 5. We then established a disease-pathway network (Figure 6). Thus, an integral way was obtained to clarify the biological processes, biological functions, and molecular mechanisms of Danshen acting on myelofibrosis. Based on the analysis, the above-mentioned preliminary major genes which existed in the top 5 terms were likely to be key or central genes in the development of myelofibrosis.

### 3.3. Obtainment and Confirmation of Potential Target by Molecular Docking

**Figure 7:**
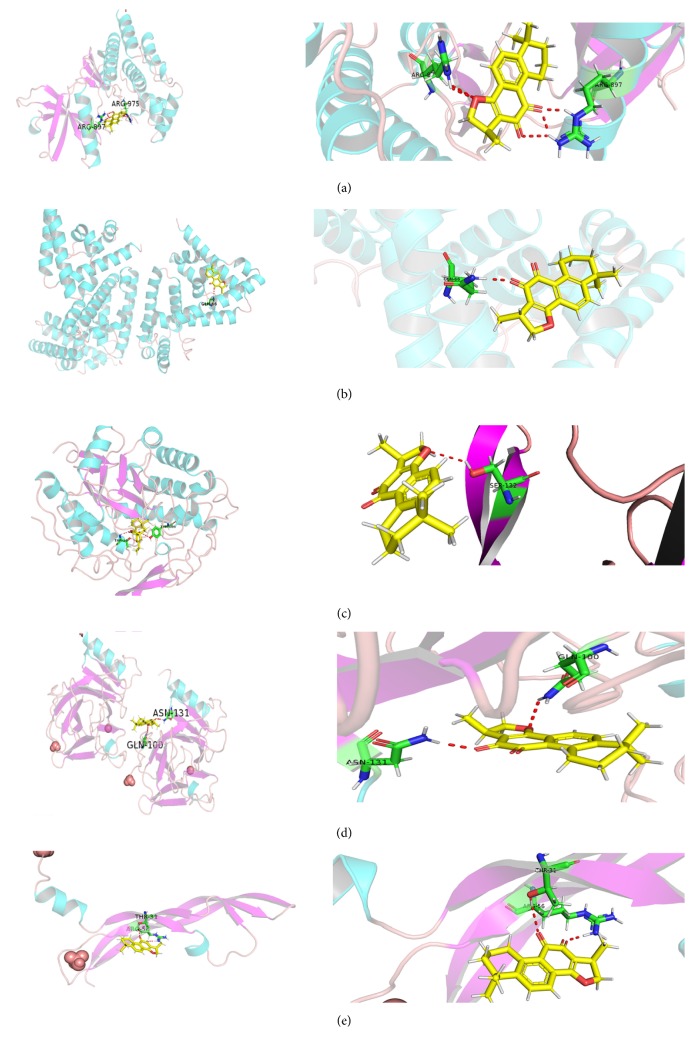
The protein-ligand of the docking simulation. (a) Cryptotanshinone and JAK2, (b) cryptotanshinone and STAT3, (c) cryptotanshinone and TNF, (d) cryptotanshinone and TP53, (e) cryptotanshinone and VEGFA.

As previously described, 5 genes (TP53, TNF, VEGFA, STAT3, and JAK2) were selected as core potential targets. Then we placed them into SYBYL-X and systemsDock for analysis of the docking potential with 20 compounds of Danshen [22]. The docking scores of the docking simulation for each target protein and ingredient are shown in Table S7. We placed the above-mentioned five pairs of gene-compounds into PyMOL software for optimization, and the specific protein-ligand interactions of docking are shown in Figure 7. Then two criteria were developed for the screening of potential core compounds: (1) docking with the 5 targets, respectively, compounds which have the highest fraction: danshenspiroketallactone (with TP53), cryptotanshinone (with VEGFA), cryptotanshinone (with STAT3), przewalskin B (with JAK2), przewalskin B (with TNF); (2) docking scores with 5 core targets were all greater than 6, including przewaquinone B, przewaquinone C, tanshinaldehyde, cryptotanshinone, iso-cryptotanshinone, tanshindiol A, and tanshinone IIB. The compound satisfying these two conditions (cryptotanshinone) was viewed as core compound which may intervene in MF by acting on core targets.

## 4. Discussion

MF is a BCR-ABL-negative clonal disorder referred to as a myeloproliferative neoplasm (MPN) [23]. Its clinical features are caused by the proliferation of clonal hematopoietic stem cells [24]. Danshen has several compounds and targets in a complex Chinese herbal medicine. Although it has been used for clinical applications such as anticancer effects for haematological malignancies, several questions regarding its mechanism of action remain [25].

In this study, we used a network pharmacology approach to analyze the potential constituent ingredients and targets of Danshen. The compound-compound target network was composed of 189 nodes (20 active compound nodes and 169 compound target nodes) and 456 edges. In the network, we found that some targets were attacked by several compounds including PTGS2, STAT3, HSP90AB1, and ACHE. This fact implied that the active chemicals of Danshen might affect these targets synergistically, virtually showing the multicomponent, multitarget, and multidisease properties of herbal medicine. Its potential effects can be highlighted by this network. The MF targets' PPI network included 103 nodes and 731 edges. Many nodes had higher degrees in this network, including TP53, TNF, VEGFA, IL8, and JAK2. The number of edges of each node was quite large (57 in TP53, 52 in TNF and VEGFA, 46 in IL8, and 40 in JAK2). Thus, these genes could be pivotal genes in the development of MF.

A compound target-MF target network was constructed with 234 nodes and 2818 edges. Danshen probably exerts its therapeutic effect on myelofibrosis by binding and regulating particular protein targets. We speculated that the top 20 nodes might be vital targets in the treatment of MF. We considered TP53, TNF, VEGFA, STAT3, and JAK2. TP53 is one of the most significant tumor inhibitor genes that plays a role in inhibiting cancer through two pathways, transcriptional-dependent activity and non-transcriptional-dependent activity in the cytoplasm of the nucleus [26]. When DNA damage is found to be irreparable, TP53 can induce apoptosis. Alternatively, if DNA is damaged, it would be activated and result in cell cycle arrest, enabling the cells to restore the damage [25]. Therefore, the status of TP53 has become an important indicator for prognosis of haematological diseases and lymphoma such as MF. TP53 abnormalities can be overcome, becoming a new target for disease treatment [26]. Osteosclerosis is the most frequently observed bone change in myelofibrosis. Various cytokines containing tumor necrosis factor (TNF) made up a complex network, mediating development, and maintenance of osteosclerosis. TNF is a pivotal mediator of cachexia and fever, exciting fibroblastic proliferation and having an effect on the proliferation of both normal and malignant cells [27]. TNF, an endogenous growth promotor, can be accompanied by bone destruction and may be related to malignant leukaemic transformation [28]. VEGFA is a member of the VEGF family, plays an important role in neurons, and is regarded as the primary inducer of the growth of blood vessels. It is completely necessary for adults who are remodelling organs and suffering diseases related to blood vessels involving tumor angiogenesis and injury healing [29]. VEGFA is also the chief exciter of both endothelial progenitor cells (EPCs) and endothelial cells (ECs) in cancer patients as well as in healthy individuals. EPCs were conducive to sustaining spleen vascularization and abnormal bone marrow that characterizes MF [30]. In the same way, recent studies have shown that angiogenesis plays an important role in the pathogenesis of haematological disorders including MPN and, therefore, MF [31]. STAT3 plays a key role in many physiological processes such as cell proliferation, differentiation, and apoptosis. As an important factor downstream of the JAK-STAT signal transduction pathway, STAT3 is involved in the pathogenesis of autoimmune diseases, myeloproliferative neoplasms, solid tumor, leukaemia, and many other diseases [32]. Various mutation sites of the STAT3 gene can cause abnormal function of STAT3 protein-related functional areas or can cause abnormal phosphorylation of STAT3, resulting in the loss of some functions of STAT3 protein and abnormal expression of related target genes, thus participating in the formation of tumors [33]. Similarly, clinical studies have found that suppressing abnormal activation of STAT3 may improve therapeutic efficacy and prognosis of tumors [34]. On the other hand, STAT3 was simultaneously targeted by 3 active chemicals of Danshen: danshenol B, cryptotanshinone, and miltionone II. Therefore, we speculate that these three active substances can treat MF by inhibiting the abnormal activation of STAT3. Mutations upstream of the JAK-STAT signalling pathway plays key roles in the pathogenesis of MF [35]. Among them, JAK is an important upstream factor of the JAK-STAT signalling pathway, including 4 family members of JAK1, JAK2, JAK3, and Tyk2 [36]. JAK is a tyrosine kinase. It is closely related to the cytoplasmic portions of receptors for pivotal hematopoietic cytokines, including thrombopoietin (TPO), erythropoietin, and granulocyte colony-stimulating-factor. Normal JAK2 functions include activation of intracellular signalling pathways after ligand binding [37]. Therefore, mutations of JAK2 are considered to be important causes of MF.

Pathway analyses were conducted using DAVID. The 14 significant pathways (P ≤ 0.05) identified in this study included several related to cancer. In fact, the “JAK-STAT signalling pathway” was one of the top three pathways. The JAK-STAT pathway is a vital effector of cytokine-dependent regulation of gene expression [34]. In particular, JAK-STAT signalling plays a central role in normal and abnormal haematopoiesis and is a key signalling pathway during myelopoiesis [38]. For the majority of MFs with JAK2 V617F, the JAK-STAT pathway arises from somatic mutations and disease development is deemed to be central to aberrant JAK-STAT signalling [39]. A study showed that the TGF-*β* signalling pathway regulated fibrosis in myeloproliferative diseases [40]. Moreover, during haematopoiesis, the TGF-*β* pathway played a vital role in stem cell quiescence and in progenitor cell differentiation [41]. TGF-*β* signalling directs normal hematopoietic stem cells (HSCs) into quiescence. It is not activated in MF, suggesting that TGF-*β* may directly and competitively suppress the growth of normal HSCs to boost proliferation of the malignant clone [42]. In summary, we speculate that Danshen may play a role in the treatment of MF through key genes acting on the JAK-STAT signalling pathway and the TGF-*β* signalling pathway.

Molecular docking is the most widely used method for calculating protein-ligand interactions, and we used the SYBL-X and systemsDock to investigate probable binding modes. The results showed that cryptotanshinone had the best activity: it docked well with 5 key targets of MF. The previous research demonstrated that the structure of aromatic ring A of tanshinones may enhance the cytotoxicity [43]. And the furano-o-quinone moiety of tanshinones is the key factor for cytotoxicity, due to its ability to produce reactive free radicals in the close vicinity of the bases to cause DNA damage [44]. The study showed that cryptotanshinone can damage cell migration and invasion in several malignancies, possibly inducing cancer cell apoptosis [45]. Actually, the previous study has shown that cryptotanshinone markedly reduces the phosphorylation of STAT3, blocks nuclear translocation, and inhibits its expression [46]. In other words, cryptotanshinone was found to be an effective anticancer agent targeting the STAT3 protein [47, 48]. The experimental results of human umbilical vein endothelial cells (HUVEC) showed that cryptotanshinone could inhibit the expression of VEGF and activate the phosphorylation of its receptor protein, thus inhibiting angiogenesis [49]. Cryptotanshinone can also alter the stability of TNF mRNA by regulating the 3'-untranslated region (3'-UTR) of TNF, thereby reducing the level of TNF expression [50]. Past studies revealed that STAT3, VEGFA, and TNF also played an important role in the pathogenesis of myelofibrosis. In addition, TP53 and JAK2 also exhibited good activity at the docking frequency. This is consistent with our previous prediction that Danshen might play a key role in MF therapy by acting on the key genes of the JAK-STAT signalling pathway and the TGF-*β* signalling pathway.

## 5. Conclusions

In summary, the current study that combines a network pharmacology method and molecular docking illuminates the molecular and pharmacological mechanisms of Danshen against MF from a systematic perspective that cryptotanshinone might play a key role in MF therapy by acting on the key genes of the JAK-STAT signalling pathway and the TGF-*β* signalling pathway. Although further experiments are needed to support our findings, the present study revealed the mechanisms of Danshen in the treatment of myelofibrosis. It provides an important basis for further study of the follow-up mechanisms and the optimization of experimental designs, so that experimental research may be more reasonable.

## Data Availability

The data used to support the findings of this study are open. The links to the databases are available at the corresponding locations in the original text.

## Supplementary Materials

Supplementary Materials Supplementary Table 1: the information of compositive compounds of Danshen. Supplementary Table 2: prediction of putative targets for compositive compounds Danshen. Supplementary Table 3: overlap of significantly targets in myelofibrosis. Supplementary Table 4: the results of topology analysis. Supplementary Table 5: re-fishing targets based on Closeness and Betweenness. Supplementary Table 6: the results of KEGG enrichment analysis. Supplementary Table 7: detailed information on molecular docking of compounds.
